# Aggressive, early resistant and relapsed mantle cell lymphoma distinct extrinsic microenvironment highlighted by transcriptome analysis

**DOI:** 10.1002/jha2.549

**Published:** 2022-10-13

**Authors:** Yannick Le Bris, Adeline Normand, Louise Bouard, Audrey Ménard, Céline Bossard, Anne Moreau, Marie C. Béné

**Affiliations:** ^1^ Hematology Biology Nantes University Hospital Nantes France; ^2^ CRCINA INSERM CNRS Université d'Angers Université de Nantes Nantes France; ^3^ Department of Pathology, Nantes University Hospital Nantes France; ^4^ Hematology Clinic Centre Hospitalier Bretagne Atlantique Vannes France; ^5^ Department of Pathology Centre Hospitalier Départemental de Vendée La Roche sur Yon France

**Keywords:** macrophages, mantle cell lymphoma, microenvironment, relapse, resistance

## Abstract

Immunotherapy strategies relying on innate or adaptive immune components are increasingly used in onco‐haematology. However, little is known about the infiltrated lymph nodes (LN) or bone marrow (BM) landscape of mantle cell lymphoma (MCL). The original transcriptomic approach of reverse transcriptase multiplex ligation‐dependent probe amplification (RT‐MLPA) was applied here to explore the expression of 24 genes of interest in MCL at diagnosis (21 LN and 15 BM) or relapse (18 LN). This allowed us to identify that at baseline, samples from MCL patients with an aggressive morphology (i.e. blastoid or pleomorphic) or a high proliferative profile, displayed significantly higher monocyte/macrophage‐associated transcripts (*CD14* and *CD163)* in LN and BM. Regarding T‐cells, aggressive MCL forms had significantly lower amounts of LN *CD3E* transcripts, yet an increased expression of cytotoxic markers in LN (*CD8*) and BM (*CD94*). A very high‐risk group with early treatment resistance displayed, at diagnosis, high proliferation (*KI67*) and high macrophages and cytotoxic transcript levels. Post‐immunochemotherapy relapsed samples revealed lower levels of T‐ and natural killer‐cells markers, while monocyte/macrophage markers remained similar to diagnosis. This study suggests that rapid analysis of MCL microenvironment transcriptome signatures by RT‐MLPA could allow for an early distinction of patient subgroups candidates for adapted treatment strategies.

## 1 INTRODUCTION

Mantle cell lymphoma (MCL) is a rare, incurable, clinically and biologically heterogeneous disease. Some patients relapse early or are immediately refractory to standard chemotherapy [1, 2], while others present an indolent course or benefit from long‐term remission after treatment. Aggressive morphology, high proliferation index [3] and some genetic abnormalities [4, 5, 6] are intrinsic features of the tumor cells associated with severe forms. However, they do not always explain differences in patient evolution in the context of intensive immunochemotherapy [1] or Bruton tyrosine kinase (BTK) inhibitors‐based regimens [7]. Moreover, little is known about the roles of the multiple actors of the immune microenvironment within the framework of MCL [8]. Information about the extrinsic environment of tumoral cells is probably of high relevance, in order to qualify how patient cells attempt to cope with the tumor or are influenced by it. In MCL, it has been shown that tumoral B‐cells depend on their environment, in particular through activation of the B‐cell receptor [9] and of CD40 by helper T‐cells [10]. Recently, myeloid cells such as tumor‐infiltrating macrophages have been identified as new potential therapeutic targets [11]. However, the impact of such cells on relapse after chemotherapy is still not well known.

The aim of the work presented here was to characterize the transcriptomic features of several actors of the intrinsic and extrinsic immune microenvironment of MCL in bone marrow (BM) and lymph nodes (LN), in a series of MCL patients with an indication for treatment. Correlations between extrinsic markers and other biological and clinical features were investigated, and their impact on outcomes was evaluated. Finally, diagnosis and post‐immunochemotherapy relapse samples were compared.

## 2 MATERIAL AND METHODS

### 2.1 Patients and samples

Forty‐five MCL patients with an indication of treatment were retrospectively included from material stored between 1999 and 2019 in the archives of the Pathology department and the tumor cell bank of the Hematology Biology department of Nantes University Hospital in France. The study was approved by the ethics committee CPP Est I and was performed in accordance with the Declaration of Helsinki.

Diagnostic criteria for MCL were revised according to the WHO 2017 classification [12]. Formalin‐fixed paraffin‐embedded (FFPE) lymph node samples (*n* = 39, 21 at diagnosis, 18 at relapse; median tumoral infiltration 80%, range 20%–90%) and frozen BM mononuclear cells (*n* = 15 at diagnosis, five at relapse; median tumoral infiltration 78%, range 13–96%) were selected (Figure S2). Patient characteristics are described in Tables S1 and S2. In the diagnosis group, 32/33 patients received treatment (immunochemotherapy *n* = 29, rituximab alone *n* = 2, chemotherapy alone *n* = 1). In the relapsed group, all samples were obtained after at least one line of immunochemotherapy except for one patient who received rituximab only.

### 2.2 Reverse transcription multiplex ligation‐dependent probe amplification

The reverse transcription multiplex ligation‐dependent probe amplification (RT‐MLPA) technique was chosen to explore the transcriptome of 24 relevant molecules. This efficient approach is of interest as it measures simultaneously the expression level of multiple genes from FFPE tumor samples not accessible to conventional microarray or more recently developed RNA sequencing approaches. To validate RT‐MLPA, expression results of intrinsic tumor‐cell genes were compared on MCL cell lines previously characterized by microarray and/or RNA‐Sequation (Figure S1). Moreover, the hyperexpression of *CCND1* and expression of *SOX11* were confirmed to be detected in the tumoral but not normal BM (Figure S3).

RNA was extracted using Maxwell blood or FFPE RNA kits (Promega, Madison, WI) depending on whether the cells were stored frozen or as FFPE blocks. cDNA was then produced by retro‐transcription using random hexamers. A panel of oligonucleotides was designed, targeting RNA from 24 intrinsic or extrinsic genes of interest. These included two MCL‐specific genes, respectively *CCND1* and *SOX11*, the proliferation indicator *MKI67*/Ki67 and probes specific to the p53 pathway *TP53*, *CDKN2A*/p16^INK4A^, *TNFRSF8/*DR5*, MDM2* and *CDKN1A/*p21. Specific probes were designed for the *CARD11* gene within the NFκB pathway as well as for one potential therapeutic target *TNFRSF8*/CD30. To assess the extrinsic tumoral microenvironment, probes were designed to explore the monocyte/macrophage compartment (*CD14* and *CD163*), T‐cells (*CD3E*/CD3 and *CD8A*/CD8), natural killer (NK) cells (CD94), the costimulation molecule CD40‐ligand (*CD40LG*/CD40L) and immune‐checkpoints (*CD152*/CTLA4, *PDC1*/PD1, *CD274*/PDL1, *PDCD1LG2*/PDL2 and *INDO1*/IDO) as well as cytokines (*CSF1*/MCSF*, IL10* and *TGFB1*). Probe sequences are detailed in Table S3. To dampen the signal of too highly expressed genes (*CCND1* and *TGFB*), competitor probes identical to the MLPA probes were designed, lacking PCR tails, and added to the mix at a 1:1 proportion.

The method published by Mareschal et al. was applied [13]. The resulting amplicons were analysed with a 3500XL Genetic Analyzer (Thermo Fisher Scientific, Waltham, MA). Hybridized probes yielded a size‐specific signal, the height of each peak being proportional to the amount of RNA initially present in the sample. The level of expression of each gene was analysed after normalizing the expression of all genes studied in the panel [13].

### 2.3 Statistics

Gene expression in different clinical or biological groups was compared with Student's or Mann Whitney/Wilcoxon tests depending on whether the variables had a normal distribution or not. The minimal level of significance considered was *p* = 0.05. Using receiver operating characteristic curve analysis, a threshold predictive of relapse for the genes tested was sought. Variables for which a threshold was detected with a *p*‐value lower than 0.05 would then be used to evaluate the impact on progression‐free survival (PFS) and overall survival (OS). PFS was measured from the date of treatment initiation to relapse, death or date of last news. OS was measured from treatment initiation to death or last reported date. Early relapse was considered when it occurred less than 1 year after treatment. Log‐rank tests and Kaplan‐Meier graphical representation, used for survival curves, and Cox logistic regression (using values statistically significant in univariate analyses) were performed using the Medcalc software (Ostend, Belgium). Primary component analysis was performed using Xlstat software (Paris, France). Free Statistics Software (v1.2.1) was used for hierarchical clustering [14].

## 3 RESULTS

### 3.1 Correlation between MCL extrinsic markers, clinical and biological features

Correlations were investigated between the expression of extrinsic micro‐environmental markers at baseline in LN and BM samples and poor prognosis biomarkers such as aggressive morphology, high proliferation index (Ki67 > 30%) and the presence of p53 disruption. The latter was defined by the detection of *TP53* mutation or p53 aberrant expression in immunohistochemistry (IHC) (Table S4).

**FIGURE 1:**
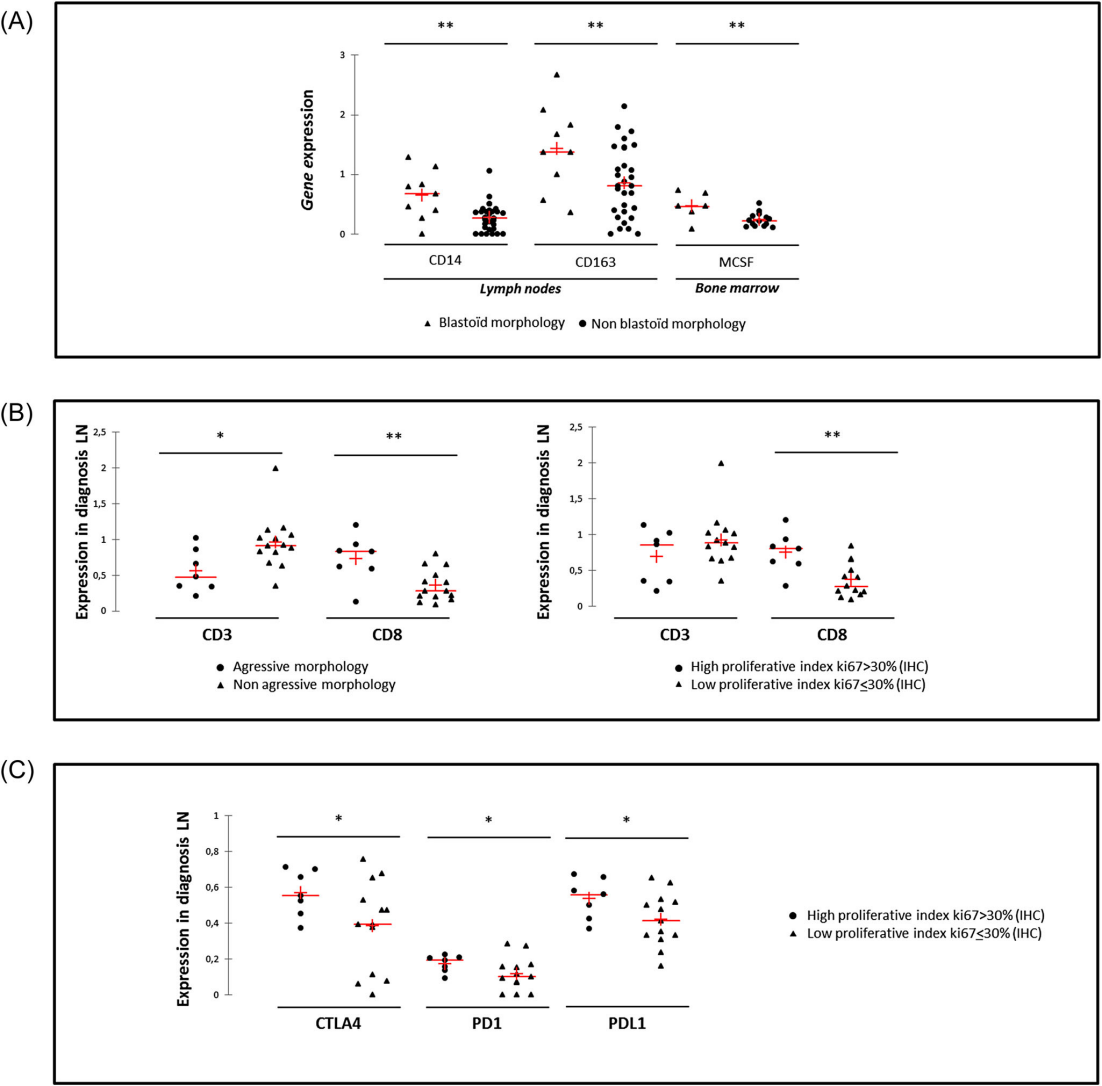
Expression profiles of extrinsic markers in the context of mantle cell lymphoma (MCL) patients with poor prognosis features. (A) Comparison of *CD14*, *CD163* and *MCSF* transcript levels measured in lymph nodes (LN) or bone marrow (BM) in the context of blastoid MCL or not. (B) Comparison between aggressive and non‐aggressive morphology tumoral LN, of the expression of T‐cell *CD3* and *CD8* markers. (C) Comparison between highly proliferative tumoral LN or not, of the expression of immune checkpoints. ** *p* < 0.001

The global expression of genes from the extrinsic microenvironment in tumoral LN and BM displayed great heterogeneity. Morphologically blastoid forms of MCL were found to be associated with higher *CD14* and *CD163* levels in LN and higher *MCSF* transcripts in BM (Figure 1A). Moreover, a higher expression of *KI67* was observed in LN (Figure S4). In LN from all aggressive forms (i.e. blastoid + pleomorphic), the amount of *CD3E* transcripts, reflecting total T‐cell infiltration, was lower than in non‐aggressive forms while *CD8* expression was increased (Figure 1B). In BM samples, there was also a trend towards higher *CD8* expression (*p* = 0.06) and a significant increase in *CD94* expression (*p* = 0.007) in cases with aggressive MCL morphology at diagnosis. Immune checkpoints (*PD1, PDL1* and *CTLA‐4*) expression was not modified according to morphology (data not shown).

Considering patients with a high proliferative index in LN, *CD8* transcripts were also statistically significantly higher in this tissue (Figure 1B) and associated with increased levels of the expression of immune checkpoints *CTLA‐4*, *PD1* and *PDL1* (Figure 1C).

**FIGURE 2:**
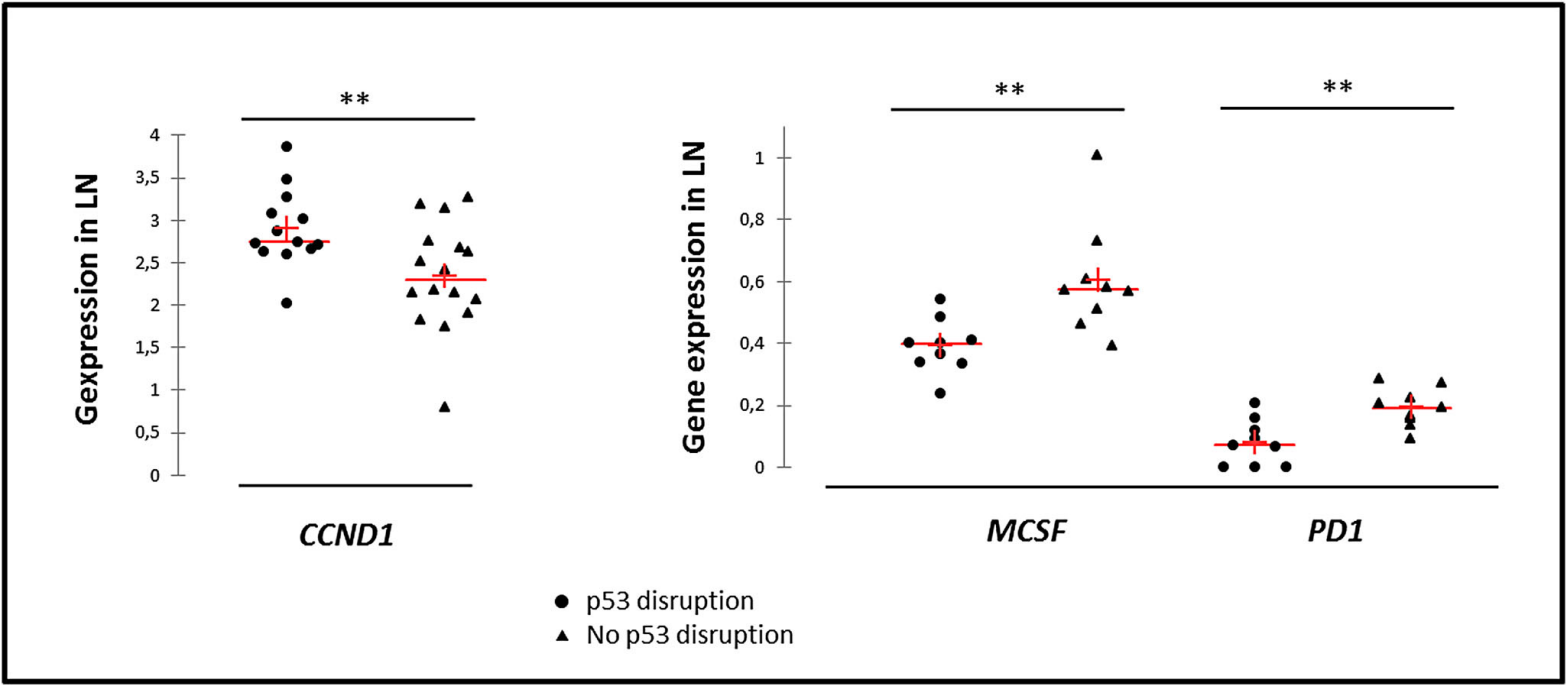
Baseline expression profiles of intrinsic and extrinsic transcripts in mantle cell lymphoma (MCL) lymph nodes according to the p53 status. P53 disruption was defined by the detection of *TP53* mutation or p53 aberrant expression in immunohistochemistry (IHC). ** *p* < 0.001

In the case of p53 disruption, the expression of *CCND1* was higher and significantly lower levels of *MCSF* and *PD1* were detected in LN at diagnosis (Figure 2) regardless of the level of tumor cell infiltration.

### 3.2 Impact of MCL intrinsic/extrinsic markers on outcomes

The prognostic value of transcripts of interest at baseline for treated patients (*n* = 32) was first considered and their impact on survival was further evaluated.

**FIGURE 3:**
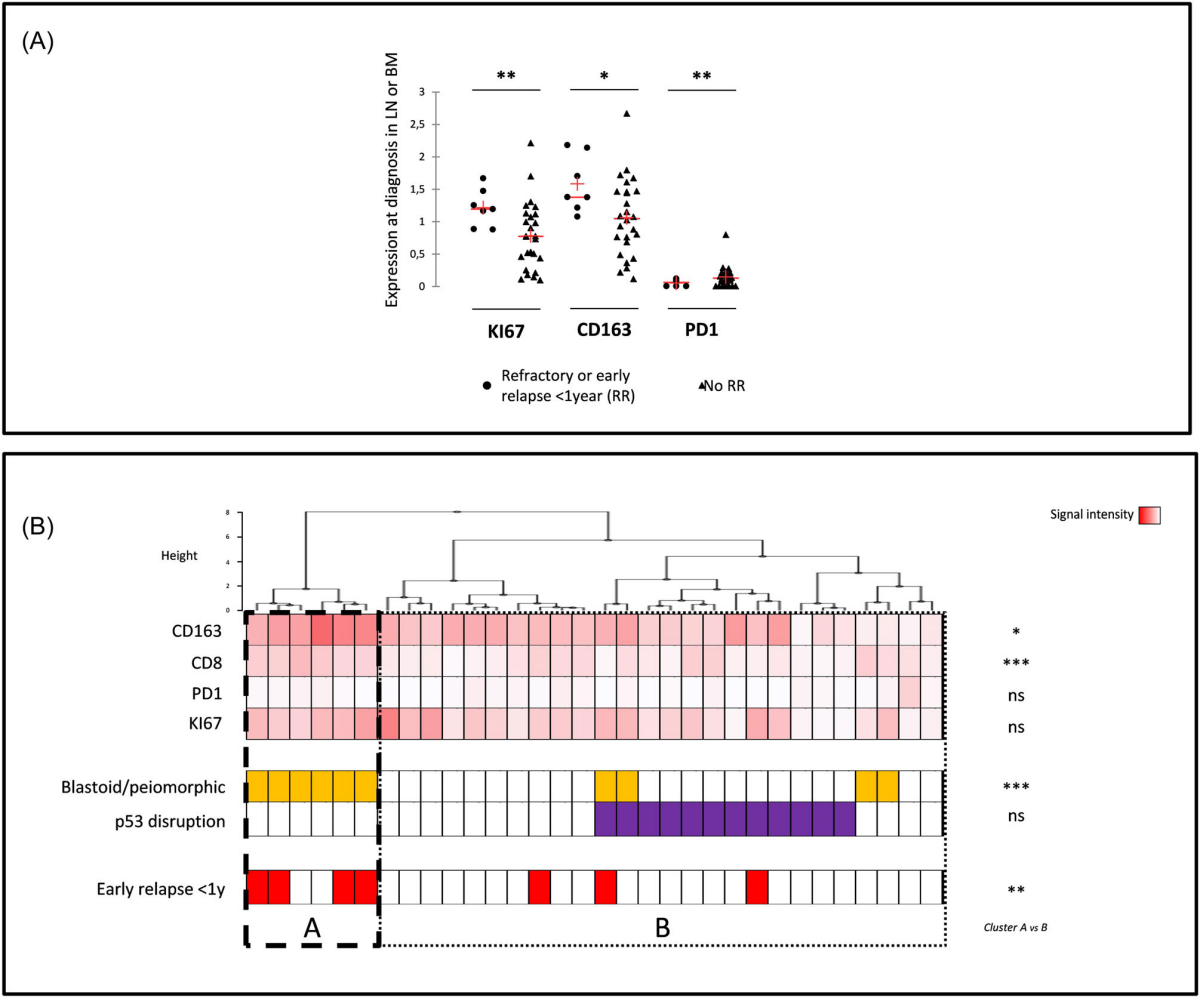
Impact of intrinsic/extrinsic gene expression on outcomes. (A) Expression profile of *KI67*, *CD163* and *PD1* in the context of refractory or early relapse < 1 year. (B) Unsupervised hierarchical cluster analysis of the expression of *CD168, CD8, PD1* and *KI67* genes (signal intensity), aggressive morphology (orange) and p53 disruption (purple) compared to refractory/early relapse (red). *p < 0.05; ** *p* < 0.001; *** *p* < 0.0001; ns: Not significant (i.e. *p* > 0.05)

The seven patients (22%) who were refractory or in early relapse (<1 year from the start of treatment) (R/R) had a more aggressive morphology (p = .002) and higher *KI67* expression in diagnosis LN and BM (pooled data) (Figure 3A). A significantly higher expression of *CD163* transcripts was also observed in these R/R patients while *PD1* levels were significantly lower than in non‐R/R patients (Figure 3A). Unsupervised hierarchical clustering of transcriptomic data (*CD163, CD8, PD1* and *KI67*) combined with morphology and p53 status segregated two clearly different groups, A and B (Figure 3B). Group A is characterized by high CD163 and CD8 expression, aggressive morphology but no p53 disruption associated with a high risk of early resistance (Figure 3B) and reduced PFS (Figure S5). Group B is more heterogeneous but interestingly comprises all patients with p53 disruption.

### 3.3 Comparison between diagnosis and relapse samples

**FIGURE 4:**
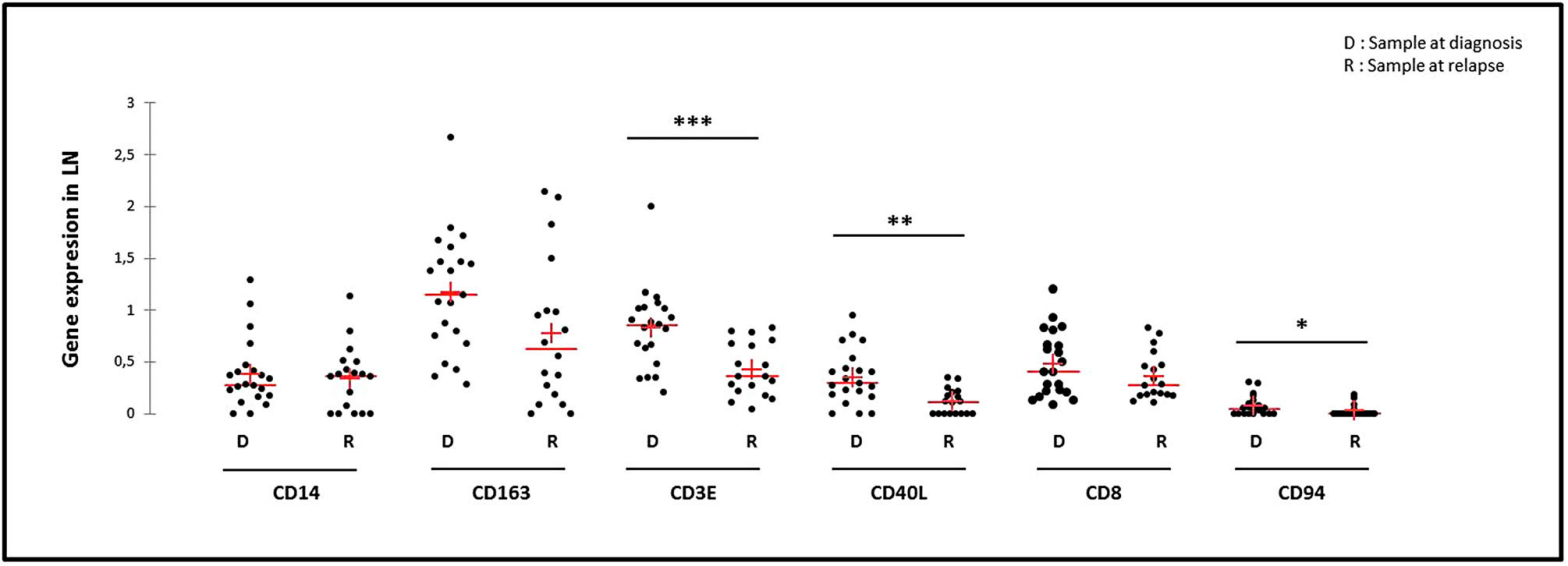
Diagnosis (D)/relapse (R) comparison profile in lymph nodes of transcript expression levels in unpaired samples of genes specific for monocyte/macrophages (*CD14* and *CD163*), total T cells (*CD3*), helper (*CD40L*) lymphocytes and cytotoxic cells (*CD8* and *CD94*). *p < 0.05; ** *p* < 0.001; *** *p* < 0.0001

Finally, a comparison of non‐paired LN sampled at diagnosis (*n* = 21) or relapse (*n* = 18) was performed. Upregulation of the levels of *CCND1* transcripts was observed at relapse while the nodal tumor load was significantly lower (Figure S6). Diagnosis/relapse comparison of extrinsic markers showed a trend for lower macrophage transcript levels (*CD94*, p = 0.052) while there was no change in *CD14* expression. Moreover, T‐cells (*CD3E* and *CD40L*) and NK‐cells (*CD94*) markers were significantly lower in relapsed LN while there was a trend for lower *CD8* transcripts (p = 0.09) (Figure 4). Paired sample analysis confirmed this decrease in T‐cells (*CD3E, CD40L* and *CD8*) and NK cells (*CD94*) transcripts at relapse (Figure S4).

## 4 DISCUSSION

The study reported here aimed at examining the expression of genes involved in the intrinsic and extrinsic microenvironment of MCL. This work used an original technique of RT‐MLPA which allowed to analyse FFPE samples with highly degraded RNA unusable with standard RNA sequencing approaches [13].

The extrinsic MCL microenvironment was thus confirmed to be highly heterogeneous. Specific alterations were observed in the context of MCL with aggressive morphology and high proliferative or p53 disruption predicting early resistance. Moreover, the immune microenvironment was found to be strongly modulated at relapse. In MCL of aggressive morphology or with active proliferation (high Ki67), *CD8* and immune‐checkpoints (*PDL1, CTLA4* and *PD1*) transcripts were higher, suggesting an impaired balance/efficacy of the anti‐tumoral immune response. In these aggressive forms, the expression levels of *CD14* and *CD163*, considered specific to M2‐like macrophages [15], were however increased in LN while *MCSF* levels were increased in BM. It has been previously shown that MCL tumoral cells could polarize macrophages toward an M2‐like phenotype via cellular contacts and soluble factors suggesting a role for this interaction in tumour aggressiveness [11].

In the context of p53 disruption, there was a lower number of *PD1* transcripts. This potentially decreased immunomodulation of tumor‐infiltrating T cells deserves to be further explored. It could be promoted by the stimulation induced by mutant neoepitopes [16, 17, 18]. In this context, we also observed a decrease in the expression of the *MCSF/*CSF1 cytokine but higher *CCND1* transcript levels independently from tumoral infiltration. *MCSF* can be expressed by tumor cells and promote macrophage differentiation towards a pro‐tumoral M2 type [11, 15]. This mechanism does not seem to function here in the presence of p53 cell cycle checkpoint abnormalities, suggesting that tumor cells grow with high levels of *CCND1* in spite of a lower level of *MCSF* expression.

In R/R patients, a potential enrichment in M2 macrophages in LN and BM via the significant increase of *CD163* appears to carry a significant value. These transcriptomic results comfort data acquired in IHC showing that CD163+ macrophages are associated with poor responses to chemotherapy and could predict resistance or early relapse [19]. As our results were derived from a retrospective cohort of patients treated with various chemotherapies, confirmation of these results in a series of patients with homogenous treatment will be a prerequisite before proposing this marker for treatment stratification.

At the time of relapse, increased *CCND1* expression was found as previously shown [20] while monocytes/macrophages were not significantly modified compared to diagnosis. Conversely, T‐cell and NK cell infiltration appeared to be lower than at diagnosis, with downregulation of the costimulation marker CD40L, important to prime cytotoxic tumor‐specific CD8 T‐cell responses [21]. This state of quantitative depression of actors of intra‐tumoral immunity at the time of relapse could be related to a persistent toxic immunosuppressive effect related to chemotherapy [22]. This demonstration of chemo‐induced alterations in the anti‐tumoral cytotoxic functions of T‐cells could be an argument to position immunotherapies at earlier treatment stages. Indeed, immunomodulatory agents, such as lenalidomide [23], would enhance immune cytotoxicity against tumor cells [24]. Moreover, CAR T‐cells for salvage treatment or relapse are another interesting alternative [25], especially through reinfused CD8 T‐cells [26].

Innovative technologies for spatial analysis of transcripts or multiplex protein expression [27] that are more cell‐specific than bulk analysis by RT‐MLPA have been recently described. Yet, RT‐MLPA is directly applicable in real life and is the only technology that was validated prospectively in a time frame compatible with clinical management [28]. This technology allows for the combined identification of *CCND1* and *SOX11* hyperexpression, which can be a diagnosis help, sometimes difficult in MCL according to the experience of the LYMPHOPATH group [29].

## 5 CONCLUSION

This study confirms, in an original transcriptomic approach, the great heterogeneity of the MCL microenvironment. Important, yet seldom described, modulations of T‐cells, NK cells, macrophages and cytokines were observed, with different profiles segregating MCL with aggressive biology markers and those with treatment resistance. Finally, RT‐MLPA was shown to be a useful, simple and easy way, in clinical practice, the exploration of intrinsic and extrinsic biomarkers of interest that could help to choose between chemo‐immunotherapy or other chemo‐free approaches in the near future.

## AUTHOR CONTRIBUTIONS

Yannick Le Bris designed the study, collected samples, performed analyses, interpreted data and wrote the manuscript; Adeline Normand collected samples and performed analyses; Louise Bouard collected clinical information; Audrey Ménard performed analyses; Céline Bossard and Anne Moreau provided and annotated samples; Marie C. Béné interpreted data and wrote the manuscript.

## CONFLICT OF INTEREST

The authors declare they have no conflicts of interest.

## FUNDING INFORMATION

The authors received no specific funding for this work.

## ETHICS STATEMENT

The CPP Est I French ethics committee approved the study on September 26, 2017 (ref:2017/50‐DC‐2014‐2206[1]).

## Supporting information

Supporting Information
