# Supplementary material for: Aggressive, early resistant and relapsed mantle cell lymphoma distinct extrinsic microenvironment highlighted by transcriptome analysis
Source: EJHaem. 2022 Oct 13;3(4):1165–71. doi: 10.1002/jha2.549 (PMC9713019; doi:10.1002/jha2.549)
Supplement: Supplementary file 1 — Supporting Information [file JHA2-3-1165-s001.docx]

**Supplemental material and data**

**Aggressive, early resistant and relapsed mantle cell lymphoma distinct extrinsic microenvironment highlighted by transcriptome analysis**

Yannick Le Bris, Adeline Normand, Louise Bouard,
Audrey Ménard, Céline Bossard, Anne Moreau, Marie C. Béné

***Methods***

***Cell lines, normal bone marrow and RNA profiling***

The MCL cell lines Mino, Maever, Z138, Rec-1 and JVM2 were obtained from the American Tissue Cell Collection (ATCC), Deutsche Sammlung von Mikroorganismen und Zellkulturen (DSMZ), graciously provided by Pr Chen Kiang or derived locally from patients. These 5 cell lines had been tested by microarray gene expression profiling (GEP) (Mino, Maever, Z138, Rec1, Jvm2) (Chiron D, et al. Blood 2016) and sequenced by RNA-Seq (Mino, Maver, Z138) and/or DGE-seq (Mino, Maever, Z138, Rec1) (Illumina, San Diego, CA) and results were compared with RT-MLPA data. Normal bone marrow (BM) (n=9) samples were collected during hip replacement surgery from healthy adult individuals.

***Pathological and immunohistochemical analyses***

All MCL LN were reviewed by experts from the LYMPHOPATH network for a verification of morphology (i.e. classical or aggressive [blastoid or pleomorphic]) and immunostaining. The latter was performed with a p53 antibody (DO7 clone, M7001292, Agilent Technologies® Santa Clara, CA) and the MiB1/Ki67 antibody (Agilent, M7240). Expression of the p53 protein was considered abnormal when it was either negative (<1% positive cells) or high (>50% positive cells) (Aukema et al. Blood 2018).

***DNA sequencing***

When enough material was available, DNA from FFPE tumors and BM was extracted with the respective Maxwell Blood DNA and RSC DNA FFPE kits (Promega). A library preparation was performed with the Qiaseq targeted DNA custom panel kit (Qiagen), composed of primers with unique molecular identifiers (UMI) targeting *TP53.* Sequencing was then carried out on Miseq (Illumina). Variant analysis was performed using a previously described academic pipeline (Dubois et al. Clin Cancer Res 2016). A minimum threshold of 100 sequenced UMIs was considered to obtain a sufficient depth of analysis, failing which the DNA was considered unusable.

***Results***

***Validation of RT-MLPA on MCL cell lines***

Gene expression profiles, assessed by RNA and DGE Seq in MCL cell lines were compared to those detected by RT-MLPA (Figure S1). Among MCL-intrinsic genes, an expected high signal was consistently retrieved for *CCND1.* Significant levels of *KI67* were observed in all cell lines while *SOX11* was expressed in Mino, Maever, Z138 and REC1 but not JVM2 as observed with other RNA quantification techniques. RT-MLPA results satisfactorily compared to RNA/DGE Seq and GEP data excepted for *CDKN1A/*p21 for which the signal was lower in RT-MLPA. This gene was subsequently not considered. Among MCL-extrinsic genes, a low baseline expression of the transcripts of *CSF1*/*MCSF* and immune-checkpoints *PD1* and *CTLA4* was observed in Mino as previously reported (Papin et al., *Leukemia* 2019; Harrington et al. *Leuk Lymphoma* 2019) as well as a low expression of *PD1* and *PDL1* in JVM2. The *PDL2* transcript was not detected in any of the cell-lines. Transcripts of *CSF1*/M-CSF, *IL10* and *TGFB*, cytokines that activate the M2 macrophage profile (Murray et al. *Immunity* 2014) were differentially expressed between cell lines. *IL10* expression was only seen in Z138 and *MCSF* in Mino as previously shown (Papin et al. *Leukemia* 2019). Finally, signals for extrinsic-specific surface markers of the monocyte/macrophage lineage (*CD14*, *CD163*), T-cells (*CD3E/CD3, CD8A/CD8, CD40LG/CD40L*) and NK cells (*CD94*) were all absent or considered as fade-in noise.

***Validation of RT-MLPA on clinical samples***

MCL BM showed a consistent high expression of the intrinsic MCL transcripts *CCND1* and *SOX11* in comparison with non-tumoral samples (Figure S2) and, of note, lower expression of *SOX11* was observed in BM samples from non-nodal MCL patients than from patients with nodal forms (Figure S2). In tumoral LN, high expression of *KI67* transcripts, measured in RT-MLPA, was correlated with strong proliferation in immunohistochemistry (IHC) (protein Ki67>30% of tumoral cells).

**Supplemental Figures**

**Figure S1 Time point (A) and type of tissues (B) of the samples selected**

**Figure S2 Validation of RT-MLPA with other transciptomic techniques**

Comparison of the performance of various techniques for the quantification of transcript levels of interest from RNA extracted from LCM lines (Mino, Maever and Z138). A normalized signal average of the classical approaches (i.e. RNAseq, DGEseq and GEP) was compared to the signal measured in RT-MLPA. The R² correlation coefficient between these two values for all the genes studied was measured.


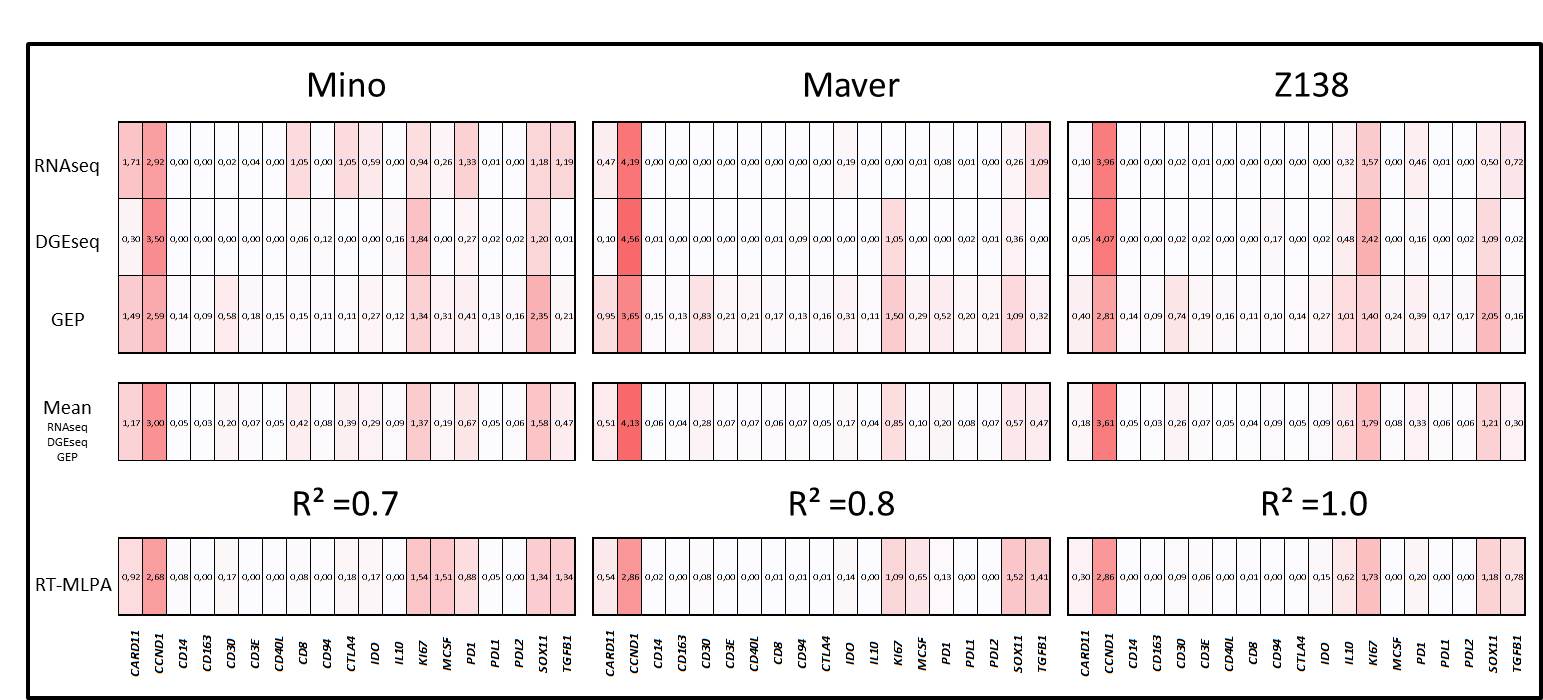


**Figure S3 SOX11 and KI67 expression in MCL samples.**

(A) Expression profile of *CCND1* and *SOX11* in tumoral and non-tumoral bone marrow (BM) samples. (B) Comparison of SOX11 expression in the BM of patients with classical clinical forms and non-nodal leukemic forms (Left). Representation of the expression level of the KI67 transcript compared to IHC (Right).


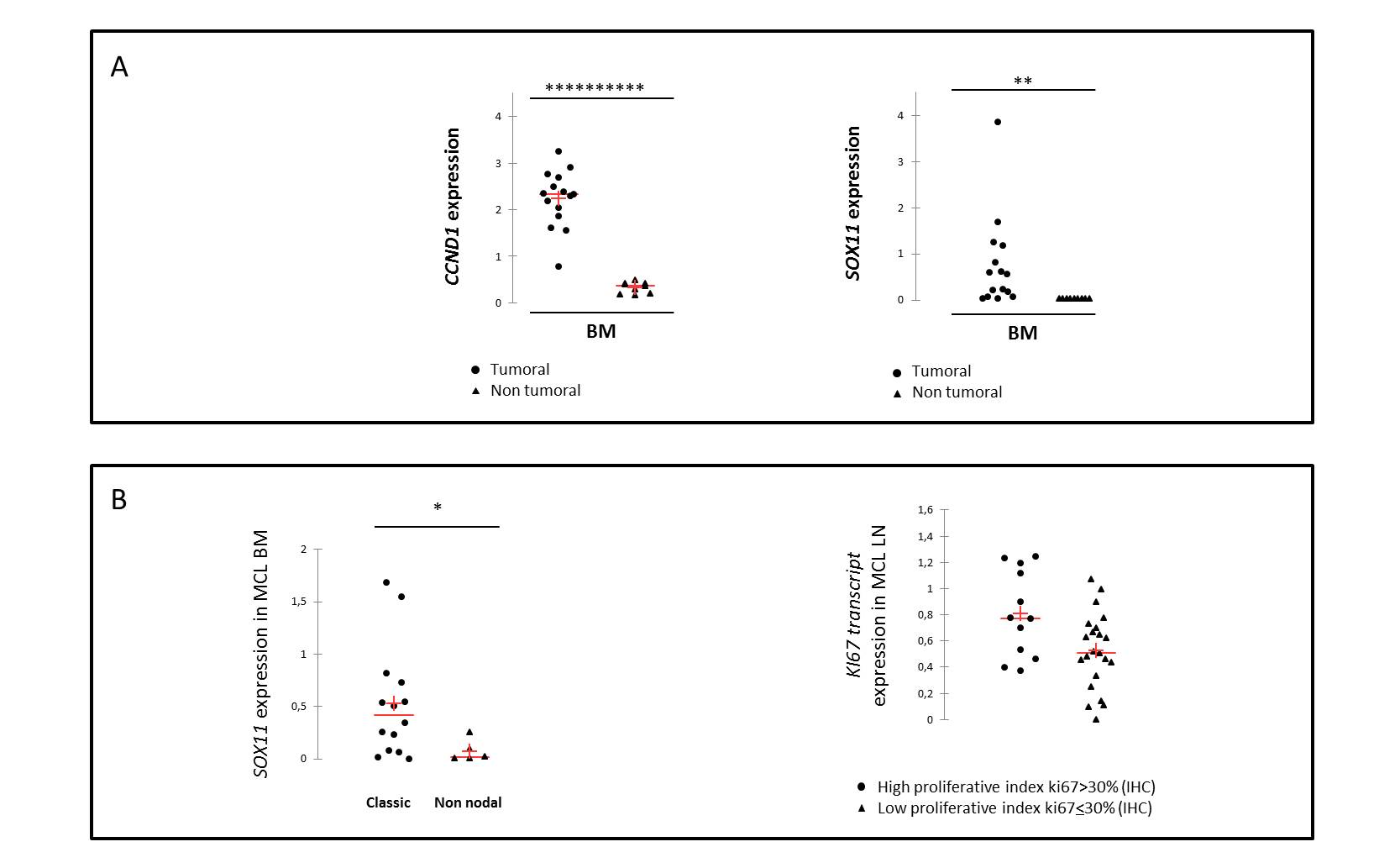


**Figure S4 KI67 transcript expression profile in lymph nodes according to morphology**

**
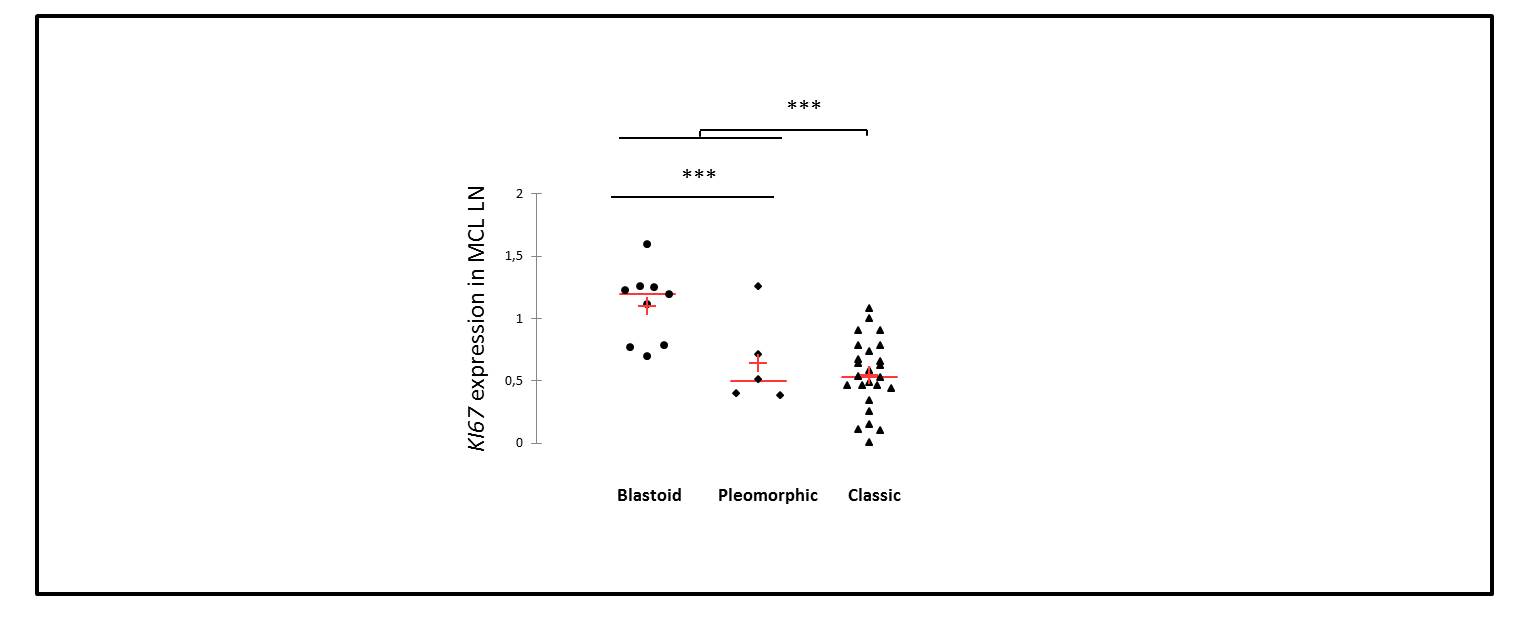
**

**Figure S5** Progression free survival (PFS) from treatment initiation to relapse, death or last follow-up (p<0.05).

**Figure S6 Diagnosis (D) / relapse (R) comparison of the tumoral cells infiltration, intrinsic and extrinsic markers.**

**A.**Diagnosis (D) / relapse (R) comparison of the tumoral cells infiltration and of *CCND1* transcript level expression in paired (right) and unpaired (left) samples. **B**. Comparison at diagnosis and relapse of transcript levels of genes specific for total T-cells (CD3), cytotoxic (CD8), NK (*CD94*) and helper (CD40L) lymphocytes in paired samples. Paired analysis was performed using bone marrow (red bars) and lymph nodes (black bars). **
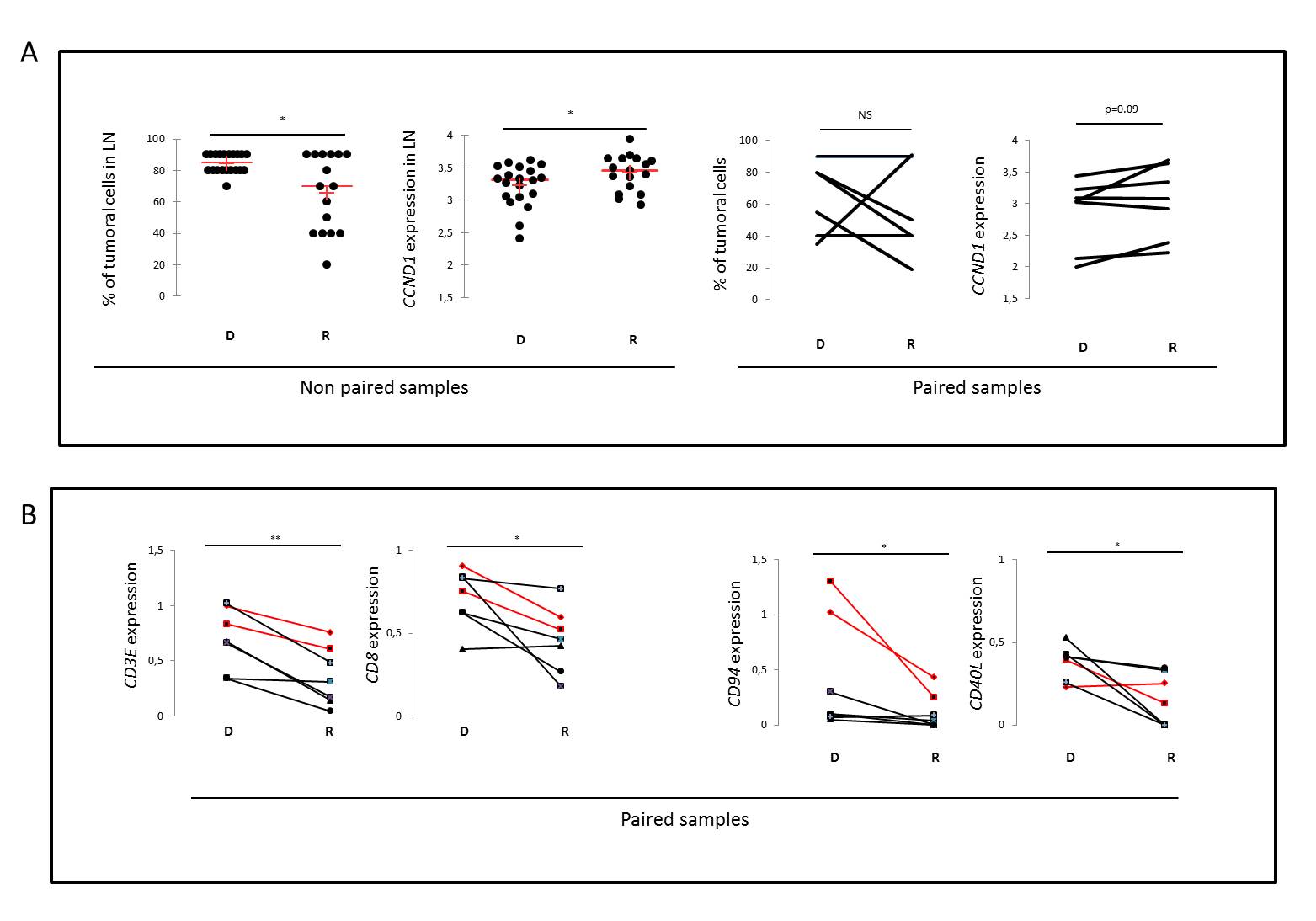
Table S1 Patient characteristics**

| **Patients** | **Diagnosis** | | **Relapse** | |  |
| --- | --- | --- | --- | --- | --- |
|  | *N=33* | | *N=21^†^* | |  |
| **Age at inclusion (years)** |  |  |  |  |  |
| Median (Min-Max) | 68 (41-88) | | 70 (47-86) | |  |
| **Gender** |  |  |  | |  |
| Male | 24 | 73% | 15 | 71% |  |
| Female | 9 | 27% | 6 | 29% |  |
| **B2m>N** |  |  |  |  |  |
| Missing | 5 |  | 7 |  |  |
| > Upper limit | 24 | 86% | 9 | 64% |  |
| **Ann Arbor** |  |  |  |  |  |
| II | 0 | 0% | 3 | 15% |  |
| III | 4 | 12% | 3 | 15% |  |
| IV | 29 | 88% | 14 | 70% |  |
| **MIPI** |  |  |  |  |  |
| Low | 4 | 12% | 1 | 5% |  |
| Intermediate | 9 | 27% | 8 | 40% |  |
| High | 20 | 61% | 11 | 55% |  |
| **Aggressive morphology** | 11 | 33% | 8 | 40% |  |
| **p53 disruption** | 9/29 | 31% | 0/13 | 0% |  |
| *TP53* mutation (LN and BM)  Abnormal p53 expression in LN | 5/17 | 29% | 4/15 | 27% |  |
| **Outcomes** |  |  |  |  |  |
| Patients treated | 32 * | 97% | - | - |  |
| Median number of treatment lines | 2 (0-5) | | - | *-* |  |
| Relapse post treatment | 19 | 57% | - | - |  |
| Refractory or relapse <1 year | 7 | 22% | - | - |  |
| Median PFS (95%CI) | 25 (16-58) | | - | - |  |
| Death | 17 | 52% | - | - |  |
| Median OS (95%CI) | 71 (32-101) | | - | - |  |
|  |  |  |  |  |  |
| * One patient with intention to treat at diagnosis withdrew consent |  |  |  |  |  |
|  |  |  |  |  |  |

*^†^* Among the 21 patients two were included at 1^st^ and 2^nd^ relapse

**Table S2 Details of patient treatments**

**Table S3 RT-MLPA probes sequences**

**Table S4 Details of p53**

d: diagnosis r: relapse

P53 disruption defined by the detection of a TP53 mutation or p53 aberrant expression in IHC

The absence of p53 disruption was defined by both the absence of a TP53 mutation and of p53 aberrant expression in lymph nodes or absence of TP53 mutation in BM
